# Quantification of EUGR as a Measure of the Quality of Nutritional Care of Premature Infants

**DOI:** 10.1371/journal.pone.0132584

**Published:** 2015-07-20

**Authors:** Zhenlang Lin, Robert S. Green, Shangqin Chen, Hui Wu, Tiantian Liu, Jingyang Li, Jia Wei, Jing Lin

**Affiliations:** 1 Department of Neonatology, The Second Affiliated Hospital and Yuying Children’s Hospital, Wenzhou Medical University, Wenzhou, China, 325027; 2 Kravis Children’s Hospital and Department of Pediatrics, The Icahn School of Medicine at Mount Sinai, New York, NY, 10029, United States of America; 3 Department of Neonatology, The First Hospital of Jilin University, Changchun, China, 130021; Hôpital Robert Debré, FRANCE

## Abstract

**Objectives:**

To develop an index of the quality of nutritional care of premature infants based on the change in weight Z score from birth to discharge and to illustrate the use of this index in comparing the performance of different NICUs.

**Methods:**

Retrospective data analysis was performed to compare the growth of premature infants born in three perinatal centers. Infants with gestational age ≤ 32 weeks who survived to discharge from 2006 to 2010 were included. Weight Z scores at birth and discharge were calculated by the method of Fenton. Using data from one NICU as the reference, a multivariable linear regression model of change in weight Z score from birth to discharge was developed. Employing this model, a benchmark value of change in weight Z score was calculated for each baby. The difference between this calculated benchmark value and the baby’s observed change in weight Z score was defined as the performance gap for that infant. The average value of the performance gaps in a NICU serves as its quality care index.

**Results:**

1,714 infants were included for analysis. Change in weight Z score is influenced by birth weight Z score and completed weeks of gestation; thus the model for calculating the benchmark change in weight Z score was adjusted for these two variables. We found statistically significant differences in the average performance gaps for the three units.

**Conclusions:**

A quality care index was developed based on change in weight Z score from birth to discharge adjusted for two initial risk factors. This objective, easily calculated index may be used as a measurement of the quality of nutritional care to rank the performance of different NICUs.

## Introduction

Linking financial incentives to the quality of care, i.e. pay-for-performance, has become a popular approach to performance improvement in health care worldwide [1]. Reimbursement for the care of premature infants in neonatal intensive care units (NICU) fits well into the pay-for-performance model because of the high cost, available databases, relative strength of research evidence, and, compared with adult settings, low incidence of pre-existing comorbidities [2]. However, at present, pay-for-performance has not been applied in the NICU setting due to lack of simple and well defined measures of quality of care. An ideal measurement of quality of care should be valid, reliable, objective, feasible, and relevant. Unfortunately, there currently is no such widely accepted, readily calculated quality care index available in NICUs [3].

Traditionally, quality measures of care for premature infants include outcome measures such as birth weight specific mortality and rates of complications of prematurity. While birth weight specific mortality is easy to define, it fails to address the surviving infants. Other neonatal outcome measures may suffer from bias or fraud, such as omitting poor outcomes in order to improve ratings without actually improving performance [2]. Further, data collection on some of the most important outcome measures for premature infants, such as neurodevelopmental status, is of necessity delayed for years or even impossible for many NICUs to ascertain, excluding their being used as the measures of quality of care for pay-for-performance reimbursement. Recently, Profit et al [4] presented a composite indicator, the baby-MONITOR, which includes a total of 9 parameters collected from each NICU, as a tool to comprehensively assess the quality of care delivered by NICUs. However, it is still too complicated to be used in each individual NICU. Furthermore, it is not applicable to an individual premature infant.

Optimizing postnatal growth is an integral component of the management strategies of preterm infants and an important health outcome measure in the NICU. Degree of extrauterine growth restriction (EUGR) therefore may be used as one of the objective measures of quality of nutritional care of premature infants. However, the definitions of EUGR are not consistent in the literature and several methods have been proposed to quantify the degree of EUGR. By analyzing the data from three regional perinatal centers, we sought to illustrate the wide differences in the incidences of EUGR resulting from the use of different definitions and to develop an objective method quantifying EUGR which may be used as a simple and fair index of quality of nutritional care in NICUs.

## Methods

Data collection: Data were retrospectively retrieved from three regional perinatal centers, one from the United States (Mount Sinai Medical Center of New York) and other 2 centers from China (labeled as China 1 and China 2). Birth weight, gestational age, discharge age and discharge weight were collected from the individual perinatal data bases or medical records on all premature infants with gestational age ≤32 weeks who survived to discharge home from 2006 to 2010. The gestational age was determined based on the best obstetric estimate with >90% of those confirmed by early prenatal ultrasound. The postmenstrual age at discharge was calculated as the sum of gestational age plus chronological age at discharge. The study was approved by the Program for the Protection of Human Subjects of the Icahn School of Medicine at Mount Sinai and the Ethics Committees of The Second Affiliated Hospital and Yuying Children’s Hospital of Wenzhou Medical University and The First Hospital of Jilin University. All patient records/information was anonymized and de-identified prior to analysis.

Statistical analysis: For each infant in the study population, weight Z scores at birth and at discharge were calculated using the LMS tables published by Fenton & Sauve [5, 6]. The Z score (Standard Deviation Score) = (X-U)/SD, where X is the individual value, U denotes mean value and SD denotes standard deviation. The several different incidences of EUGR were calculated using the definitions proposed by others in the literature and compared between the three groups. Continuous variables were compared by analysis of variance (ANOVA) between the groups and post hoc comparisons between each group were done using the Bonferroni test. Categorical variables were compared by the chi square test.

Change in weight Z scores from birth to discharge was selected as the quantitative measure of EUGR for each patient. Arbitrarily using the Mount Sinai group as the reference NICU, we developed a multivariable linear regression model of change in weight Z score from birth to discharge as a function of birth weight Z score and completed weeks of gestation at birth. Then, for each baby in the study a benchmark value of change in weight Z score from birth to discharge was calculated using the baby’s birth weight Z score and completed weeks of gestation in the model equation. We then define the performance gap for each baby as the difference between the baby’s observed value of change in weight Z score and this calculated benchmark value (performance gap = observed change in weight Z score—benchmark change in weight Z score). The mean value of the performance gap for the babies in each NICU, which by definition must be zero for the reference NICU, was defined as the quality care index for that unit, and the values of the three NICUs were compared by ANOVA and the Bonferroni test. Data are presented as means and standard deviations, and P<0.05 is considered as statistically significant. All statistical analysis was performed by using IBM SPSS 20 (IBM, Armonk, NY).

## Results

### The basic demographic characteristics of the three groups at birth and at discharge

There were a total of 2444 infants (662 babies from the Mount Sinai NICU, 894 from the China 1 NICU, and 888 from the China 2 NICU) with gestational age ≤32 weeks admitted into the 3 NICUs. However, only 1,714 infants who survived to discharge home (605 babies from the Mount Sinai NICU, 562 from the China 1 NICU, and 547 from the China 2 NICU) were included in the data analysis. The babies not included in the analysis either died before discharge or were transferred out for medical, social, or economic reasons. The basic demographic characteristics of the three groups are shown in the Table 1. There were no differences between the birth weight Z scores for the three groups, indicating the growth status at birth was similar for 3 groups despite the gestational age and birth weight were significantly different. There were also significant differences between the three groups for age at discharge, discharge weight, and weight Z score at discharge.

**Table 1:** The basic demographic characteristics of the three groups at birth and at discharge.

	Mt. Sinai (n = 605)	China1 (n = 562)	China 2 (n = 547)	p (Mt. Sinai vs. China1)	p (Mt. Sinai vs. China 2)	p (China 1 vs. China 2)
	Mean ± SD	Mean ± SD	Mean ± SD			
Gestational Age, weeks	29.4 ± 2.4	30.4 ± 1.7	30.8 ± 1.3	0.000	0.000	0.004
Birth Weight, grams	1355 ± 409	1531 ± 345	1589 ± 352	0.000	0.000	0.029
Birth Weight Z Score	-0.106 ± 0.721	-0.031 ± 0.729	-0.030 ± 0.911	0.313	0.314	1.000
Discharge Age, weeks	7.7 ± 4.7	6.1 ± 3.3	5.6 ± 2.6	0.000	0.000	0.041
Discharge PMA, weeks	37.6 ± 2.9	36.5 ± 2.3	36.4 ± 2.2	0.000	0.000	0.640
Discharge Weight, grams	2486 ± 529	2155 ± 389	1973 ± 325	0.000	0.000	0.000
Discharge Weight Z Score	-1.042 ± 0.724	-1.345 ± 0.782	-1.647 ± 0.897	0.000	0.000	0.000
Change in Weight, grams	1130 ± 737	624 ± 492	384 ± 354	0.000	0.000	0.000
Change in Weight Z Score	-0.939 ± 0.637	-1.314 ± 0.619	-1.617 ± 0.744	0.000	0.000	0.000
PMA: Postmenstrual Age						

### The incidences of EUGR were significantly different among the three NICUs

The incidence of intrauterine growth restriction (IUGR) defined as weight Z-score at birth <-1.28 (birth weight <10^th^ percentile) and the incidences of EUGR according to three different definitions are presented in the Table 2. The three different definitions of EUGR were: 1. Discharge weight Z score < -1.28 (discharge weight < 10^th^ percentile); 2. Change in weight Z score < -1.28; 3. Change in weight Z score < -2.0. As shown in the Table 2, there were significant differences between the three groups regardless of which definition of EUGR was employed. However, none of these three measures of EUGR adjusts for potential confounding factors present at the time of birth prior to admission of the babies to their respective NICUs.

**Table 2:** Incidences of intrauterine and extrauterine growth restriction of the three groups.

	Mt. Sinai	China 1	China 2	p (Mt. Sinai vs. China 1)	p (Mt. Sinai vs. China 2)	p (China 1 vs. China 2)
	(n = 605)	(n = 562)	(n = 547)			
Birth Weight Z Score < -1.28	34 (5.6%)	24 (4.3%)	47 (8.6%)	0.346	0.051	0.003
Discharge Weight Z Score < -1.28	213 (35.3%)	293 (52.1%)	356 (65.2%)	0.000	0.000	0.000
Change in Weight Z Score < -1.28	154 (25.5%)	258 (45.9%)	363 (66.5%)	0.000	0.000	0.000
Change in Weight Z Score < -2	27 (4.5%)	70 (12.5%)	135 (24.7%)	0.000	0.000	0.000

Weight Z score < -1.28 = <10^th^ percentile

### Changes in weight Z score is influenced by the initial birth weight Z score and gestational age

**Fig 1:**
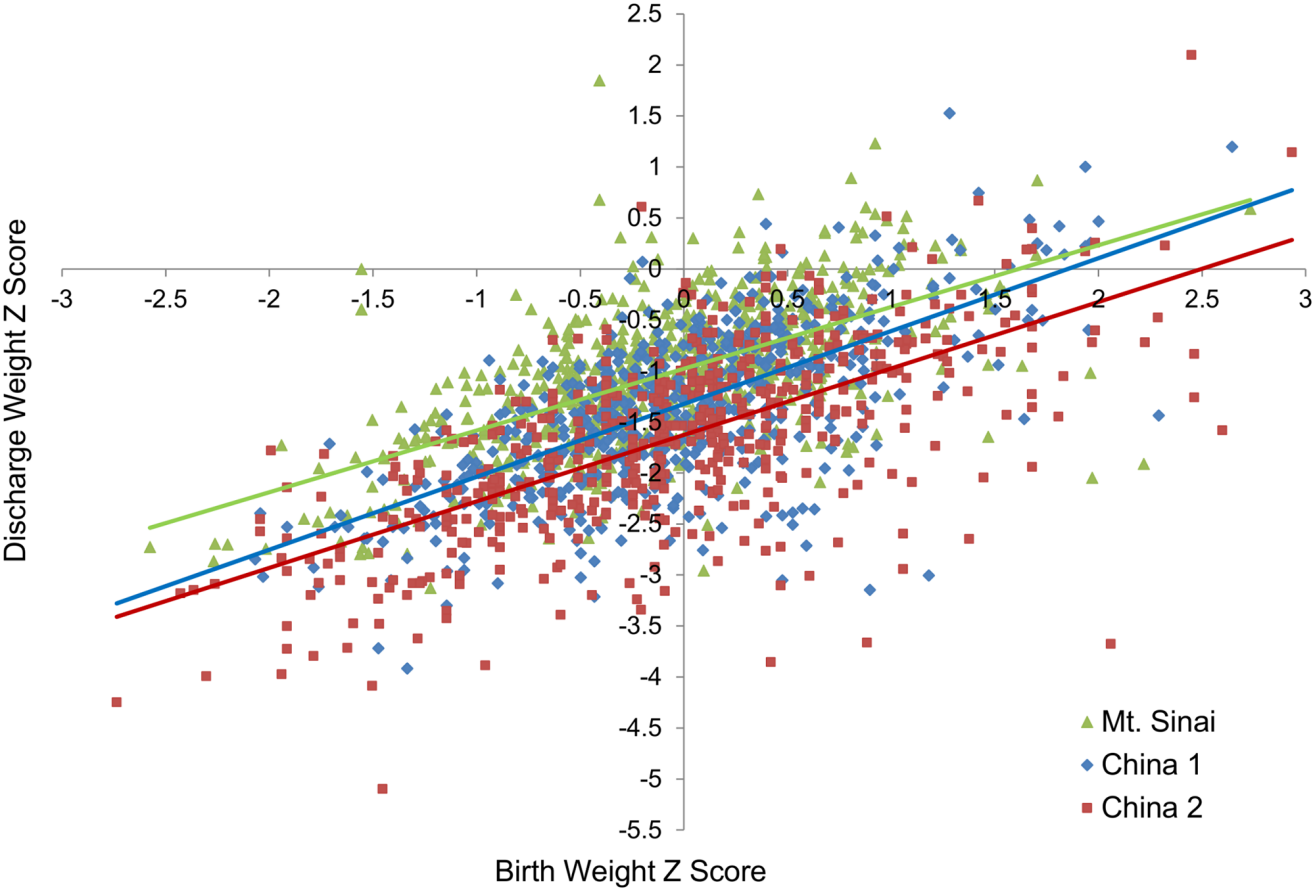
Discharge weight Z score as a function of birth weight Z score for all three NICUs. The regression lines for the three NICUs are for Mount Sinai, y = 0.61x–0.98 (R^2^ = 0.36, p<0.001); for China 1, y = 0.71x–1.32 (R^2^ = 0.44, p<0.001); and for China 2, y = 0.65x–1.62 (R^2^ = 0.44, p<0.001). Thus, the y intercepts for all three NICUs are <0 and the slopes for all three NICUs are <1, reflecting EUGR.

**Fig 2:**
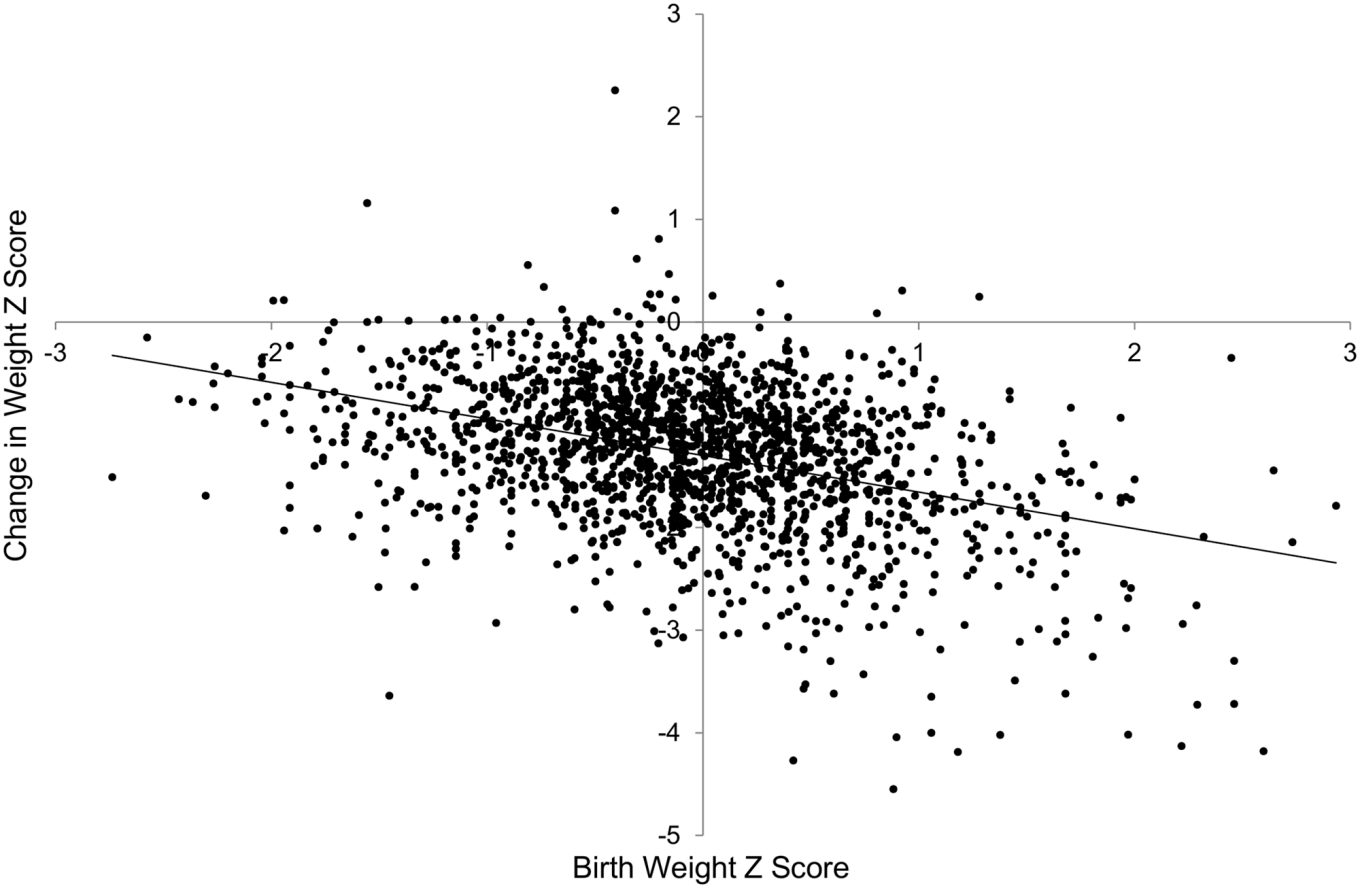
Change in weight Z score from birth to discharge vs. birth weight Z score for the reference NICU (Mount Sinai). Regression line: y = -0.387x-0.980, R^2^ = 0.192 (p<0.001).

**Fig 3:**
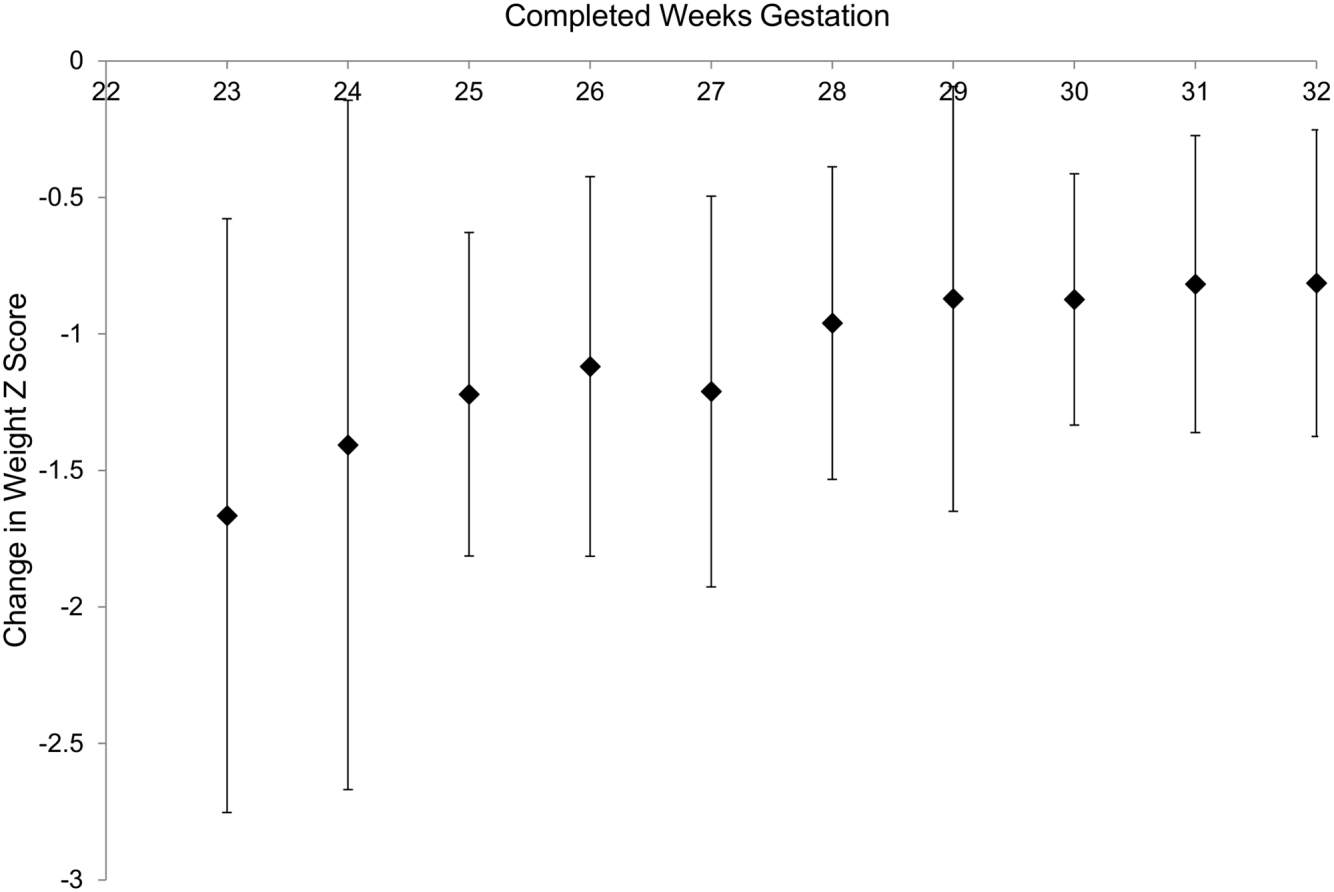
Change in weight Z score from birth to discharge (mean ± SD) vs. completed weeks gestation at birth for reference NICU (Mount Sinai). Regression line y = 0.066x-2.867, R^2^ = 0.063 (p<0.001).

In attempting to develop a quality care index, we would like to adjust for confounding factors which are beyond the control of NICU staffs. The relationship of weight Z score at discharge and birth weight Z score is presented in the Fig 1. As the figure illustrates, the weight Z score at discharge was lower than the birth weight Z score in the large majority of babies, indicating near universal growth restriction in the NICU. However, in order to compare the growth failure in different NICUs over a given time period, it would be preferable to adjust for pre-existing confounders rather than to simply use weight Z score at discharge. To design a model which would allow calculation of a benchmark value for each infant, we arbitrarily chose the Mount Sinai babies as the reference group. As expected and illustrated in Figs 2 and 3 using data from the reference NICU, the change in weight Z score from birth to discharge is influenced by the birth weight Z score and the gestational age at birth.

### Changes in weight Z score after adjusting for birth weight Z score and gestational age may be used as a measure of the quality of care with which to compare NICUs

**Fig 4:**
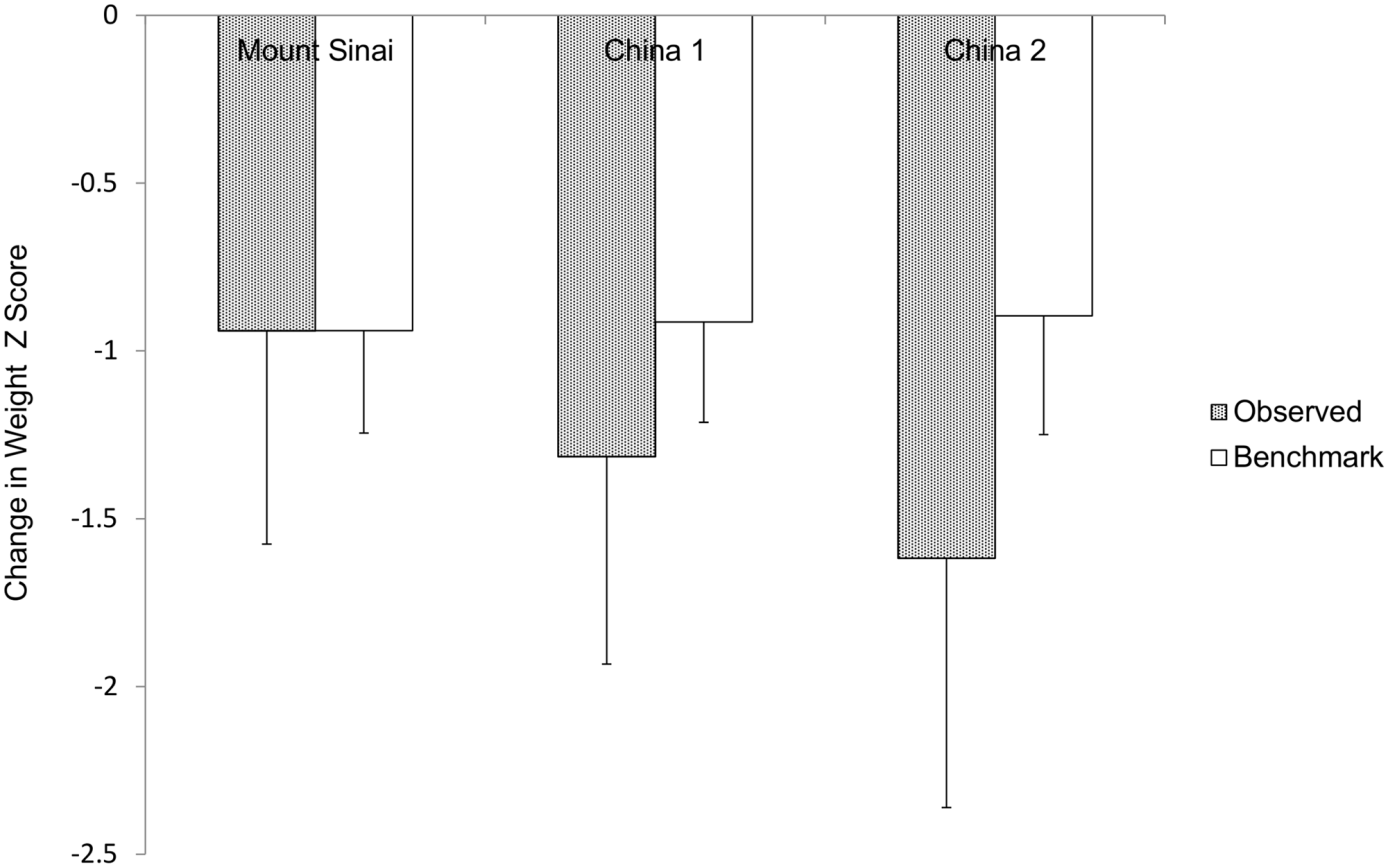
Observed and benchmark values of change in weight Z score from birth to discharge for the three NICUs. The benchmark values for the three NICUs do not differ significantly (p>0.05). The observed values for the two Chinese NICUs differ from that of Mount Sinai, from each other, and from their benchmark values (p<0.001 for all the comparisons). Per model design, the observed and benchmark values are the same for the Mount Sinai NICU.

As delineated above, birth weight Z score and completed weeks of gestation at birth affect the degree of EUGR defined as change in weight Z score from birth to discharge in the reference population. In order to adjust for the effects of these two preexisting factors on the degree of EUGR, a multivariable linear regression model was constructed using the Mount Sinai NICU as a reference group, expressing change in weight Z score as a function of birth weight Z score and completed weeks of gestation. The equation generated is: benchmark value for change in weight Z score = -2.486 + (0.051 x completed weeks gestation) + (-0.364 x birth weight Z score). The R^2^ for this model is 0.23 so over 75% of the variability of the changes in weight Z score from birth to discharge is unexplained and, at least in part, is a function of the postnatal care in the NICU. This equation was then used to calculate the benchmark values of change in weight Z score for each baby in all three groups using their birth weight Z scores and gestational ages. The mean values of benchmark and observed changes in weight Z score from birth to discharge for the three NICUs are shown in Fig 4. We define the difference between the observed and benchmark values of change in weight Z score from birth to discharge for each baby as the performance gap for that infant. The average performance gap for the babies in a particular NICU serves as the quality care index for that unit. By design of the model, the mean benchmark and observed values for the Mount Sinai reference NICU are the same. The quality care index (mean±SD) for China 1 and China 2, -0.400±0.552 and -0.721±0.654 respectively, are negative compared to that of the reference NICU, which is zero by design (0.000±0.559) (p<0.001). In addition China 2 NICU’s value is significantly more negative than that of China 1 (p<0.001).

## Discussion

We show in the current study that, regardless of the definition, the incidence of EUGR differed between the three NICUs. We also illustrate that the degree of EUGR as measured by the change in weight Z score from birth to discharge in our reference population is influenced by birth weight Z score and gestational age. We develop a linear regression model using data from a reference NICU which allows calculation of benchmark values for change in weight Z score adjusted for birth weight Z score and completed weeks of gestation in babies from other NICUs. We define the performance gap for each baby in those NICUs as the difference between observed and benchmark changes in weigh Z score. We define the quality care index as the average performance gaps in that NICU. We demonstrate that the three NICUs differ significantly using this quality care index.

The multiple morbidities that premature infants experience during their NICU stay often lead to postnatal growth failure [7, 8]. It has been shown previously that the incidence of EUGR increases with decreasing gestational age [9]. Variation in postnatal growth between NICUs has been noted; furthermore, consistent with our observations, the difference cannot be explained completely by factors present at birth such as birth weight and gestational age [10, 11]. It has been suggested that variation in practices account for the largest difference in growth failure between different NICUs [11]. This indicates that the degree of EUGR may be used as a measure of the quality of nutritional care in individual NICUs. Indeed, for the three NICUs used as examples in this study, significant differences in a measure of EUGR persist even when adjusted for the preexisting factors of gestational age and birth weight Z score.

Postnatal growth failure may have prolonged effects on outcomes, such as delaying the recovery from chronic lung disease, rate of later physical growth, and, possibly, cardiovascular and metabolic disorders in adult life [12]. Optimal postnatal growth of a premature infant is also associated with a better long-term neurologic outcome [13, 14]. While the goal of nutritional management of premature infants is to achieve postnatal growth velocity that mimics intrauterine growth rates [15], nutrient intakes needed to meet this goal may be difficult to achieve in very premature infants. However, overall weight gain in premature infants can be improved with the implementation of an educational program to raise caregivers’ awareness of nutritional practices associated with the better weight-gain outcomes [16, 17]. Additionally, good NICU care which leads to lower incidences of prematurity-related complications such as late onset sepsis, necrotizing enterocolitis, and chronic lung disease also will improve the postnatal growth [18–22]. Thus, as the result of both improved caretakers’ education and enhanced risk reductions, better postnatal growth adjusted for preexisting risk factors, may reflect a better quality of care of premature infants.

EUGR has been defined by some as discharge weight less than the 10th percentile [8, 19, 20], but this definition fails to consider the prenatal growth status and gestational age of the baby. Others have used the various degrees of decrease in weight Z score from birth to discharge to define EUGR [6, 10, 18, 22]. Although this method does account for the prenatal growth status, it does not adjust for gestational age. Furthermore, it also relies on an arbitrary cut off point for the definition of EUGR. Thus, we have developed a simple method of quantifying EUGR which can be calculated from readily available data, takes into account both prenatal growth status and gestational age, is a continuous number, and can be used as a quality care index.

The major limitation of this study is the use of only two factors to adjust the model of EUGR. Certainly, the use of other prenatal factors such as gender, race, multiple gestations and the use of prenatal steroids would improve our model. However, adding those factors into the model will definitely increase the complexity and work load for reliable data collection. Furthermore, it has been shown before that adding those factors contributes very little to the explanation of growth differences of extremely premature infants between NICUs [11]. Although measures of initial illness severity such as the 5 minute Apgar score or the Score for Neonatal Acute Physiology (SNAP) might be included in the model as confounding factors since they may be predictive of outcomes including postnatal growth [11, 23], they in fact reflect, at least in part, the quality of initial care rendered to the premature baby either in the delivery room or in the NICU. The simplicity of adjusting for only gestational age and birth weight Z score is attractive and the index we have defined is easy to determine with data which are readily available in any perinatal data base or easily extracted from medical records. However, we are not suggesting using Mount Sinai as a reference nursery for future studies. Rather, we are illustrating how one NICU can be used as a benchmark for other NICUs or for management in that NICU itself. For example, we are currently comparing our current growth data at Mount Sinai to the data from 2006–2010 to see if our nutritional management has improved.

Obviously, birth weight specific mortality rate should always be considered if we apply this simple method to compare the quality of care among NICUs. Many other aspects of care, i,e, the incidence of bronchopulmonary dysplasia (BPD) and the neurodevelopmental outcome should be considered as well. Furthermore, unadjusted comparisons of outcomes among NICUs involving large numbers of transferred babies may be invalid unless the entire network is included as a whole unit. For example, Olsen et al [24] have shown that variation in the prevalence and timing of transfers out of NICUs lead to differential censoring of outcome data, such as growth, that may significantly bias the comparisons between sites. Therefore, for NICUs whose population has a significant portion of outborn infants, our simple model may reflect the quality of nutritional care at both the institution of birth and the referral center. Similarly for NICUs that transport infants to community hospitals to complete their NICU course, the quality of nutritional care in both institutions is reflected in our quality care index.

In summary, we have used data from three different NICUs to illustrate that a reference NICU can be used to determine benchmark values of change in weight Z score from birth to discharge for babies in other NICUs. A performance gap for each baby can readily be calculated as the difference between the observed and benchmark values. The average performance gap for babies in a NICU over a given time period may serve as a quality care index for that unit. In addition to birth weight specific mortality rate, incidence of BPD, and neurodevelopmental outcomes, this objective, easily calculated quality care index might be used as one of the measurements of care quality to rank the performance of different NICUs or to compare quality of nutritional care over time within one unit.
